# Plant Growth‐Promoting Rhizobacteria Isolated From Natural Habitats Promote the Growth of *Elymus sibiricus* and Enhance Its Resistance to Abiotic Stress

**DOI:** 10.1002/pei3.70106

**Published:** 2025-12-26

**Authors:** Ruiqi Liang, Li Zhong, Zeyao Huang, Weixia Wang, Guangxin Lu, Tingheng Zhu

**Affiliations:** ^1^ College of Biotechnology and Bioengineering Zhejiang University of Technology Hangzhou Zhejiang Province China; ^2^ China National Rice Research Institute Hangzhou Zhejiang Province China; ^3^ College of Agriculture and Animal Husbandry Qinghai University Xining China

**Keywords:** abiotic stress, *Bacillus mycoides*, *Elymus sibiricus*, IAA, PGPR, plant growth promoting

## Abstract

*Elymus sibiricus* L., a perennial tufted herbaceous species native to the Qinghai‐Tibet Plateau, serves as a crucial forage resource and plays a vital role in ecological restoration of degraded vegetation. To discover beneficial microorganisms that promote its growth and enhance its stress resistance, this study isolated two novel Plant Growth‐Promoting Rhizobacteria (PGPR) strains, *Bacillus mycoides* GN‐1 and *Bacillus* sp. MQ‐5 from rhizospheric soils of wild *E.* *sibiricus* populations on the Qinghai‐Tibet Plateau. Both strains demonstrated plant growth‐promoting traits, including indole‐3‐acetic acid (IAA) synthesis and siderophore production. In pot experiments, MQ‐5 treatment increased aboveground fresh biomass by 25.90% (*p* < 0.05), increased root biomass by 270.51% (*p* < 0.001), while GN‐1 treatment increased 19.29% (*p* < 0.05) and 54.38% (*p* < 0.001), respectively, compared to the control. Furthermore, MQ‐5 alleviated salinity and drought stress in *E. sibiricus* , highlighting its potential for improving forage productivity and resilience in fragile high‐altitude ecosystems.

## Graphical abstract

Two novel PGPR strains, *Bacillus mycoides* GN‐1 and *Bacillus* sp. MQ‐5, which were isolated from soils on the Qinghai‐Tibet Plateau, were found to enhance the growth of *Elymus sibiricus* through the synthesis of indole‐3‐acetic acid (IAA) and the production of siderophores. These strains were found to elevate aboveground biomass by up to 25.9% and root biomass by 70.5%. These strains mitigate the effects of salinity and drought, demonstrating their potential to increase forage productivity and the ecological resilience of fragile, high‐altitude ecosystems.

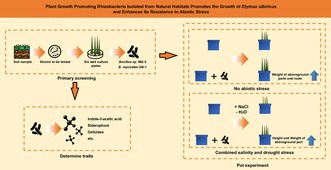

## 1 Introduction

*Elymus sibiricus* L. is a perennial herbaceous plant of the genus *Elymus* spp. in the family Gramineae, with a wide range of distribution (Li et al. 2023; Ntakirutimana et al. 2019; Wang et al. 2018). *E. sibiricus* has the dual functions of soil and water conservation and as livestock feed: *E. sibiricus* has strong adaptability, developed roots, cold and drought resistance, which is the dominant species in the alpine meadow and grassland community of the Qinghai‐Tibet Plateau, widely used in the construction, restoration and improvement of the grassland located in Qinghai‐Tibet Plateau (Xie et al. 2017; Zhang et al. 2015, 2018; J. Zhou 2023); *E. sibiricus* has high nutritional value, good palatability, abundant leaves, soft grass quality and is rich in protein and starch. It is an excellent green forage that is palatable to livestock (Huang et al. 2020; Peng et al. 2022; Wang et al. 2020).

However, due to global climate change and increased human resource exploitation in recent years, ecological problems such as glacier retreat, soil erosion, land desertification and salinization, and a sharp decline in biodiversity in the Qinghai‐Tibet Plateau are becoming increasingly serious (Yue et al. 2024). The rise in global temperatures has caused permafrost degradation in the Qinghai‐Tibetan Plateau. The cold, dry climate has exacerbated soil desertification. Permafrost degradation and desertification cause salt to migrate from deep within the soil to the surface through water transpiration, resulting in soil salinization. At the same time, soil salinization, thawing of frozen soil, and overgrazing have exacerbated soil erosion (Yang et al. 2023). Grassland animal husbandry, as an important component of agriculture and rural economy in the Qinghai‐Tibet Plateau, has been severely affected (L. Zhou 2022). As a result, the growth environment of *E. sibiricus* has gradually deteriorated, soil degradation and salinization have intensified, and biomass and grain yield have decreased. Restoration and establishment of natural grasslands face enormous challenges. Therefore, promoting the yield of *E. sibiricus* and improving its resistance to stress have become urgent issues for ecological restoration of the Qinghai‐Tibet Plateau.

Plant growth promoting rhizobacteria (PGPR) are a type of bacteria that are recruited to colonize on the surface and inside of roots, or the rhizosphere soil, which can promote plant growth (Compant et al. 2019; Parray et al. 2016). PGPR can inhibit plant pathogenic microorganisms, enhance plant immune systems, increase nutrient intake, enhance tolerance to abiotic stress, promote plant adaptation to environmental changes, and have a wide range of beneficial effects on plant health. Plants recruit and provide nutrients to surrounding microorganisms through root exudates (Amine et al. 2018; Gustavo et al. 2021; Guttman et al. 2014; Kihyuck et al. 2021). Common genera of PGPR include *Alcaligenes*, *Arthrobacter*, *Azospirillum*, *Bacillus*, etc. (Habtamu and Mulugeta 2021). PGPR can promote plant growth by regulating the concentration of phytohormones and the activity of 1‐aminocyclopropane‐1‐carboxylic acid (ACC) deaminase, increasing the content of antioxidant bioactive compounds, secreting extracellular polysaccharides, and regulating the expression of stress‐resistance related genes and proteins (Luo et al. 2019; Priya et al. 2021; Zeng et al. 2024).

The application of PGPR has great significance for sustainable green agriculture. Excessive application of chemical fertilizers leads to soil acidification and salinization, destruction of microbial communities, inhibition of soil organic matter degradation, and water eutrophication (Li, Jia, et al. 2017; Singh 2018). In comparison, PGPR has more advantages when applied in agriculture. It is safer to the environment and human health, easily degraded in soil, and is unlikely to inhibit plant growth (Tabassum et al. 2017).

At present, there are few studies on PGPR of *E. sibiricus* . Li, Song, et al. (2017) treated *E. sibiricus* seeds with the endophytic fungus *Epichloë*, which increased the dry weight of 42‐day‐old plants. Li (2020) treated *E. sibiricus* seedlings with a PGPR mixture, which significantly promoted plant growth and improved resistance to Fusarium head blight. Li (2023) used bioinformatics methods to analyze the dominant microorganisms in the rhizosphere of *E. sibiricus* . By high‐throughput sequencing of the *nifH* gene, they obtained four strains of *Azospirillum brasilense* , *Nostoc commune* , *Methylococcus capsulatus* , and *Pseudomonas hunanensis*, which have nitrogen‐fixing properties. After preparing a mixture and treating the seeds of *E. sibiricus* , the plant height increased by 65.22% compared to the control group after 14 days, and the protein content of the leaves increased by 111.52%.

These studies had narrow sample collection sources, small sample sizes, small testing and screening scales, and did not obtain highly effective PGPR of *E. sibiricus* . They also did not explore the alleviating effects of PGPR on drought and salinization stress that *E. sibiricus* is susceptible to suffer. The aim of this study is to collect samples from multiple habitats and use an in vivo screening method to obtain PGPR that can promote the growth of *E. sibiricus* and alleviate abiotic stress.

## 2 Material and Method

### 2.1 Collection of Microbial Samples

Both Qinghai Province and Gannan Tibetan Autonomous Prefecture in Gansu Province have a wide distribution of *E. sibiricus* . Microbial soil samples were collected from the rhizosphere of *E. sibiricus* and from Tibetan region and numbered. Each sample collected independently to prevent cross‐contamination and stored at 4°C.

### 2.2 Reagents Used in Experiment

CMC‐Na medium: CMC‐Na 5 g/L, yeast extract 2 g/L, KH_2_PO_4_ 0.5 g/L, MgSO_4_ 0.5 g/L. Prepare with deionized water, mix evenly, and divide into Erlenmeyer flasks, each containing 100 mL, and add 20 g/L agar powder.

Salkowski reagent: Weigh 1.125 g of FeCl_3_ solid and dissolve in 75 mL of deionized water, measure 147 mL of 98% concentrated sulfuric acid and add slowly with stirring, and add water to make up to 250 mL. Allow the solution to cool and store in a brown bottle protected from light.

LB medium, MS medium, and CAS medium are produced by Qingdao Hopebio Biotechnology Co. Ltd.

### 2.3 Strains Isolation

Weigh 10 g of soil sample separately, containing 90 mL of sterile water with several glass beads, culture at 200 rpm for 30 min at 30°C. Remove and allow to stand for 1 min, extract the top liquid and obtain a 10× diluted leachate sample diluted with a gradient of sterile water to obtain a series of 10–10^4^‐fold dilutions. Extract 100 μL of each bacterial fluid separately, spread on an LB plate using a sterilized glass spreader and incubate at 30°C for 48 h.

Select the plate with colonies in good growth condition and uniform distribution. Select the strains with good growth condition and obviously different colony morphological characteristics from others, spread on LB plates to separate and culture, pass for several times until the pure colonies are obtained, preserve in 30% glycerol solution and store, inoculate on LB medium plate for activation before each experiment.

### 2.4 Primary Screening of *E. sibiricus* PGPR Strains

Inoculate each strain to be tested to test tubes containing 5 mL LB medium and cultured at 200 rpm at 30°C for 24 h. After centrifugation to collect the bacteria, wash with sterile water, centrifuge again to collect the bacteria, add sterile water to adjust the bacteria culture to OD_600_ = 1.

The primary screening of *E. sibiricus* PGPR was carried out in plastic six‐well culture plates. The *E. sibiricus* variety R‐3b for experiment was provided by Qinghai Grassland Improvement Experiment Station. Sand is sieved through 20 mesh sieve, washed and sterilized, each well was filled with 5 g. Put 10 seeds them into an EP tube containing 1 mL bacterial solution to soak for 30 min, and the control group was soaked in sterile water. Add the soaked seeds into wells containing the bacterial solution and the seeds were distributed evenly on sand. Cultured in an artificial light incubator at 28°C, light exposure for 12 h per day, add 3 mL of sterile water every day. On Day 7, retain 8 seedlings per well and add 1 mL of MS medium. Germination data were recorded daily. After 15 days, harvest *E. sibiricus* seedlings, measure plant height, root length and fresh weight of aboveground parts and roots. Each treatment included three replicates, with 3 wells in each group and 8 plants in each well, for a total of 24 plants.

### 2.5 Evaluation of the Ability of PGPR to Alleviate Abiotic Stress in *E. sibiricus*

The seeds of *E. sibiricus* were treated with YJ‐5, MQ‐3, MQ‐5, GN‐1, GN‐2, and GN‐3, the treatment and culture conditions were same as above. Add NaCl solution to each well on the 5th day to control the salt content of sand to 0.4% as salinity stress; Reduce the added sterile water to 1.5 mL daily from the 8th to 15th day as drought stress; Reduce the added sterile water to 1.5 mL daily from the 8th to 15th day, and add NaCl solution to each well on the 5th day, control the salt content of sand to 0.4% as combined stress of salinity and drought. On the 7th day, retain 8 seedlings per well and add 1 mL MS medium. Germination data were recorded daily. After 15 days, harvest *E. sibiricus* seedlings and measure the plant height, root length and fresh weight of aboveground parts and roots. Each strain treatment included three replicates.

### 2.6 Pot Experiment for Re‐Screening

Inoculate MQ‐5 and GN‐1 in LB medium for 12 h each and collect the bacteria, wash with sterile water and adjusted to OD_600_ = 10. Sow 16 seeds of *E. sibiricus* in seedling pots (10 × 10 × 10, cm) containing nutrient soil, grown in an artificial light incubator at 28°C, light exposure for 12 h per day, and supplemented with 50 mL of sterile water every 3 days. At 5 days after germination, retain 12 seedlings in each pot, add 7 mL of bacterial solution to the soil around the seedling roots, add sterile water to the control group. Every 7 days, add 20 mL of MS medium to each pot. Each strain treatment included three replicates. Germination data were recorded daily. After 35 days, harvest *E. sibiricus* and measure plant height, root length, fresh weight, and dry weight of aerial parts and roots.

### 2.7 Pot Experiment Under the Combined Stress of Salinity and Drought

The experimental procedure was the same as above. At 5 days after germination, add NaCl solution to each well on the 5th day to control the salt content of the sand to 0.4%; supplemented with 50 mL of sterile water every 6 days; other method is the same as in chapter 2.5. Germination data were recorded daily. After 35 days, harvest *E. sibiricus* , measure plant height, root length, fresh weight and dry weight of aerial parts and roots.

### 2.8 Molecular Identification of Strains

Use universal primers 27F (5′‐AGTTGATCMTGGCTCAG‐3′) and 1492R (5′‐GGTTTACCTTGTTACGACTT‐3′) to amplify the V1‐V9 region of the 16S rRNA gene sequence. The PCR reaction system is shown in Table S1, and the PCR program is shown in Table S2. The amplified product was cloned and sequenced (Hangzhou Tsingke Biotechnology Co. Ltd.), BLAST analysis and alignment were performed on the obtained sequence in the GeneBank database, and a phylogenetic tree was constructed using MEGA 11.0.

### 2.9 Determination of PGPR Traits of *E. sibiricus*

#### 2.9.1 IAA Production Capability

Extract 100 μL of bacterial solution (OD_600_ = 1), inoculate into a test tube containing 5 mL LB medium with 0.1 g/L L‐tryptophan, and culture at 200 rpm for 48 h at 30°C. After extracting the bacterial solution and centrifuging, add 100 μL of the supernatant to a 96‐well plate, and add 100 μL of Salkowski solution to each well. Allow the plate to stand in the dark for 30 min at room temperature, measure the OD_530_ value using a microplate reader, and calculate the IAA yield of the strain from the standard curve.

#### 2.9.2 Siderophore Production Capability

Add 100 μL of 0.2 mol/L 2,2′‐bipyridyl ethanolic solution to LB medium (50°C), mix evenly and pour the plate. Add 3 μL of bacterial solution (OD_600_ = 1) dropwise to the plate and incubate at 30°C for 2 days. The Chrome azurol S (CAS) detection medium was melted (50°C) and covered on the LB plate, placed at 30°C for 4 h. Measure the colony diameter and the discoloration circle diameter, denoted by “*d*” and “*D*”, and calculate the value (Schwyn et al. 1987).

#### 2.9.3 Cellulose Degradation Ability

Add 3 μL of bacterial solution (OD_600_ = 1) dropwise to the CMC‐Na medium plate and incubate at 30°C for 3 days. Stain with 1 mg/mL Congo Red solution for 15 min, rinse with deionized water, and soak in 1 mol/L NaCl solution for 30 min. Measure the colony diameter and the clear zone diameter, denoted by “*d*” and “*D*”, and calculate the value (Sarsaiya et al. 2018).

### 2.10 Statistical Analysis

Data were analyzed using SPSS 24.0; a *t*‐test was used to analyze the significance of the data, with *p* < 0.05 indicating a significant difference; graphs were created using GraphPad Prism 8.

## 3 Result

### 3.1 Strains Isolation

A total of 74 microbial strains were isolated, including 19 strains isolated from soils in Qinghai Province and natural grasslands in Gannan, Qinghai Tibet Plateau, 38 strains isolated from saline–alkali soil and salt‐tolerant plant roots in Zhangye, Gansu Province, 14 strains isolated from soil collected from rice fields in Fuyang, Zhejiang Province, and 3 strains isolated from camel feces in the Badain Jaran Desert, 13 soil microorganisms were stored in the laboratory and a total of 87 strains for primary screening of PGPR of *E. sibiricus* .

**FIGURE 1:**
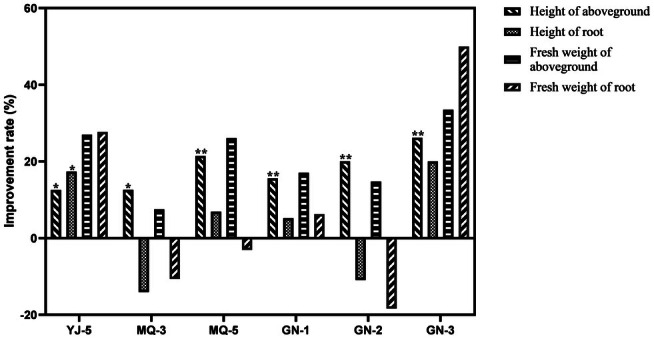
Effect of primary screening strains on biomass of *E. sibiricus* *p < 0.05; **p < 0.01; no mark: not significant.

Plant height, root length, aboveground, and fresh root weight of *E. sibiricus* are important indicators to measure the growth promoting ability of PGPR, especially the plant height and aboveground biomass. The results showed that some strains showed a promoting effect on the growth of *E. sibiricus*. Among them, the aboveground fresh weight of *E. sibiricus* treated with GN‐3, YJ‐5, MQ‐4, MQ‐5, and YJ‐3, respectively, was significantly increased, accounting for 5.75% of the tested strains; QSH‐2, YJ‐3, MQ‐1, YJ‐4, and GN‐3 significantly increased the fresh weight of roots, accounting for 5.75% of the tested strains; YJ‐5, MQ‐3, MQ‐5, GN‐1, GN‐2, and GN‐3 had a significant growth promoting effect on the height of *E. sibiricus* , which increased by 12.55%, 12.60%, 21.45%, 15.62%, 20.06%, and 26.19%, respectively, compared to the control group, and the fresh weight of aboveground parts increased by 26.99%, 7.53%, 26.13%, 17.08%, 14.76%, and 33.49%, respectively, of which GN‐3 had the most obvious growth promoting effect (Figure 1). These growth promoting strains were mainly isolated from the habitat soil of *E. sibiricus* collected from the Qinghai Plateau and the saline alkaline soil of Zhangye, Gansu Province. In addition, some strains have growth inhibitory effects. Among them, 3 strains significantly reduced the plant height of *E. sibiricus*, representing 3.45% of the strains tested. There are relatively few strains that can significantly promote the root of *E. sibiricus* .

The 16S rRNA gene of the PGPR strain of *E. sibiricus* obtained from the primary screening was amplified by PCR and sequenced, and BLAST sequence alignment was performed, and identified according to the similarity and colony morphological characteristics. The genus and source are shown in Table 1; the 16S rRNA sequences are shown in Table S3.

**TABLE 1:** Strains, genus, and source.

Strain	Genus	Source
YJ‐5	*Kocuria rosea*	Saline–alkali soil, collected from Zhangye, Gansu Province
MQ‐3	*B. cereus*	Soil of *E. sibiricus* grassland, collected from Qinghai Plateau
MQ‐5	*Bacillus* sp.	
GN‐1	*B. mycoides*	Soil of *E. sibiricus* grassland, collected from Gannan, Gansu
GN‐2	*B. subtilis*	
GN‐3	*Brevibacillus laterosporus*	

The phylogenetic tree of GN‐1 and MQ‐5 was constructed using Mega 11.0. GN‐1 has the highest homology with *B*. *fungoides* PAMC 29434 (OR029679), and MQ‐5 has the highest homology with *B*. *fungoides* NOK 111 (ON287188) (Figure S1).

### 3.2 Effect of PGPR Strains on Abiotic Stress Alleviation in *E. sibiricus*

To further screen the PGPR strains screened in the primary screening for their ability to alleviate abiotic stress in *E. sibiricus* , seedlings were grown in six‐well plates and subjected to salinity or drought treatments.

#### 3.2.1 Effect of PGPR Strain on the Growth of *E. sibiricus* Under Salinity Stress

*Elymus sibiricus* under saline stress in seeds treated with MQ‐3, MQ‐5, GN‐1 and GN‐2 was increased by 18.53%, 16.25%, 14.18% and 13.96%, respectively, as compared with the control, and the plant growth promotion effect was remarkable (Figure S2).

The mean fresh weight of aboveground parts of *E. sibiricus* under salinity stress increased by 28.2% (*p* < 0.01), 22.67% (*p* < 0.05), and 21.34% (*p* < 0.05), respectively, compared with the control after treatment with MQ‐5, MQ‐3, and GN‐1, respectively. There was a significant effect on the increase in fresh weight of the aboveground portion.

The average fresh weight of the root under salinity stress was increased by 30.78% and 23.73% after MQ‐5 and GN‐3 treatments, respectively, and had a significant effect on the increase of the fresh weight of the root (Figure S3). We selected four strains GN‐1, GN‐2, MQ‐3, and MQ‐5, which had better growth promoting effect on *E. sibiricus* under salinity stress.

#### 3.2.2 Effect of PGPR Strain on the Growth of *E. sibiricus* Under Drought Stress

After treatment with MQ‐5, the average plant height of *E. sibiricus* under drought stress was 12.07% higher than that of the control group, and had a significant effect on the increase of plant height (Figure S4).

All 5 strains did not show significant effect on the average fresh weight of aboveground part of *E. sibiricus* under drought stress (Figure S5). Among the four strains, GN‐1 and MQ‐5 had the best effect on alleviating drought stress.

#### 3.2.3 Effect of PGPR Strain on the Growth of *E. sibiricus* Under Combined Salinity and Drought Stress

None of the five strains showed a significant effect on *E. sibiricus* under combined salinity and drought stress. However, after treatment with MQ‐5 or GN‐1, biomass showed a wider band improvement (see Figures S6 and S7). GN‐1 and MQ‐5 had the best effect in alleviating the stress of *E. sibiricus* among the 6 PGPR strains obtained from the primary screening. GN‐1 and MQ‐5 could significantly increase the biomass of *E. sibiricus* under salinity stress, and MQ‐5 could significantly increase the plant height of *E. sibiricus* under drought stress. Therefore, we selected GN‐1 and MQ‐5 for further verification by pot experiment.

### 3.3 Pot Experiment

Compared to the six‐well plate culture conditions, the environment in the pot experiment is closer to the natural habitat of *E. sibiricus* ; and the culture time is longer, which is helpful to further verify the growth promoting effect of GN‐1 and MQ‐5.

#### 3.3.1 Effect of PGPR Strains on the Growth of *E. sibiricus* in Pot Experiment

**FIGURE 2:**
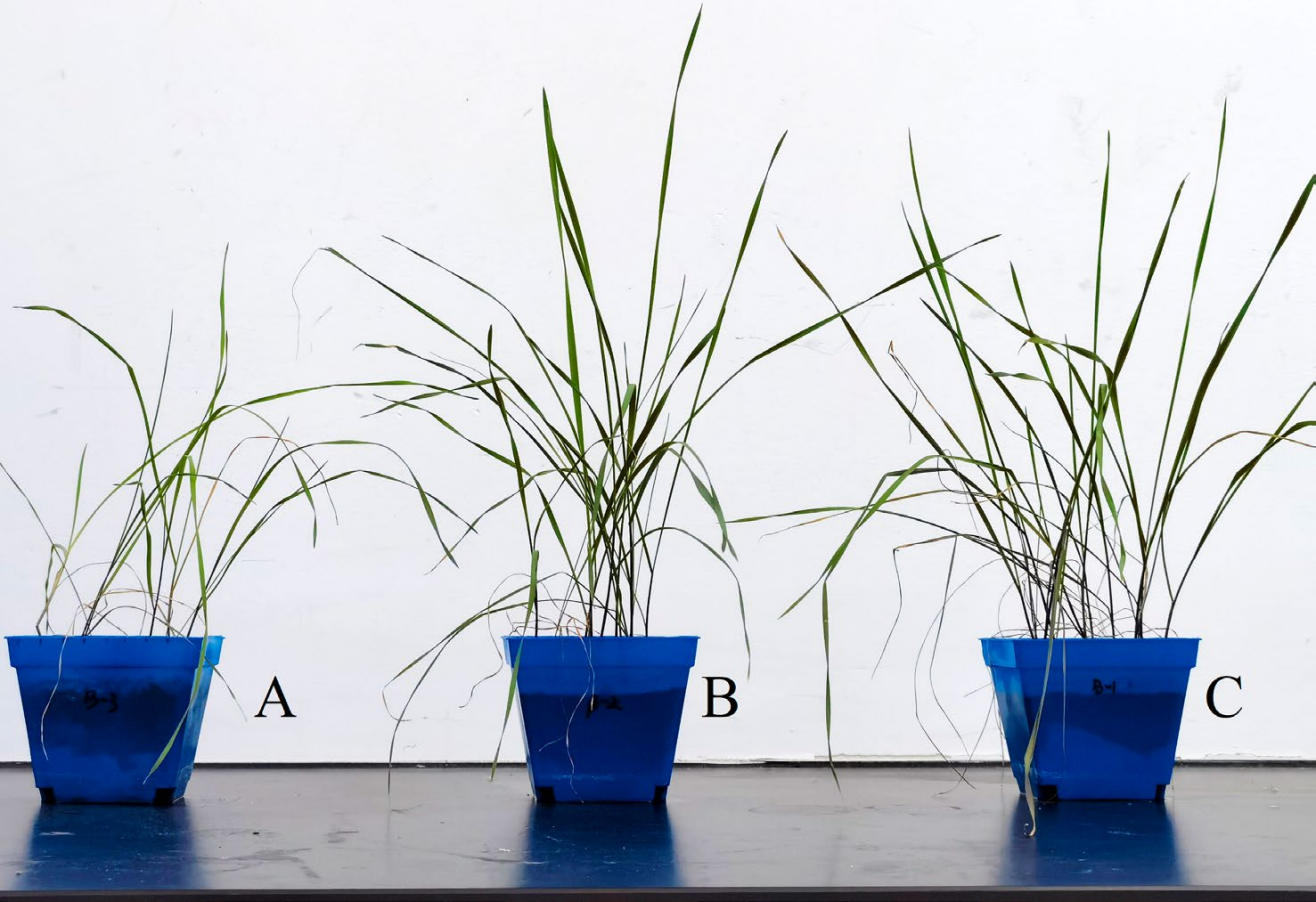
Effect of PGPR on *E. sibiricus* in pot experiment. (A) control group, (B) GN‐1 treatment group, (C) MQ‐5 treatment group.

After MQ‐5 and GN‐1 were inoculated into the root of potted *E. sibiricus* , respectively, the average plant height was increased by 14.86% and 4.73%, respectively; after treatment with MQ‐5 and GN‐1, the average root length was increased by 7.23% and 3.01%, respectively. MQ‐5 has a growth‐promoting effect on the plant height of *E. sibiricus* , and GN‐1 and MQ‐5 have no obvious effect on the root length (Figure S8 and Figure 2A).

MQ‐5 and GN‐1 could significantly increase the fresh weight of aboveground parts and roots, and MQ‐5 had the best effect on the growth promotion of *E. sibiricus* . After treatment with MQ‐5 and GN‐1, the average fresh weight of aboveground part of *E. sibiricus* was increased by 25.90% and 19.29%, respectively; After treatment with MQ‐5 and GN‐1, the average fresh weight of the root was increased by 70.51% and 54.38%, respectively (Figure 2B).

The average dry weight of aboveground part of *E. sibiricus* was increased by 21.13% after treatment with MQ‐5. The average dry weight of the root was increased by 62.91% and 48.93% after treatment with MQ‐5 and GN‐1, respectively (Figure 2C). Both MQ‐5 and GN‐1 could increase the aboveground dry weight of *E. sibiricus* , and could significantly increase the dry weight of root. MQ‐5 had the best growth promoting effect on the dry weight of *E. sibiricus* .

#### 3.3.2 Effect of PGPR Strains on the Growth of *E. sibiricus* Under Combined Salinity and Drought Stress in Pot Experiment

**FIGURE 3:**
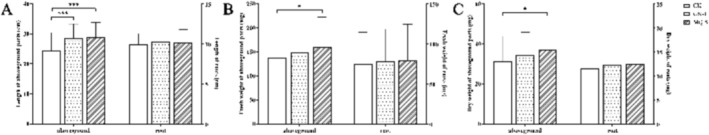
Effect of PGPR on (A) plant height and root length (B) fresh weight of aboveground parts and roots (C) dry weight of aboveground parts and roots of *E. sibiricus* under salinity and drought stress in pot experiment. *p < 0.05; ***p < 0.001; No mark: not significant.

After MQ‐5 and GN‐1 were inoculated into the root of potted *E. sibiricus* , respectively, the average plant height wasincreased by 18.02% and 16.89% (Figure 3A). Both MQ‐5 and GN‐1 could increase the plant height of *E. sibiricus* under combined salinity and drought stress with significant effect, and MQ‐5 had the best growth‐promoting effect; however, both MQ‐5 and GN‐1 had no significant effect on the root length of *E. sibiricus* .

After treatment with MQ‐5, the average fresh weight of aboveground part of *E. sibiricus* was increased by 17.04% (Figure 3B). Both MQ‐5 and GN‐1 could increase the fresh weight of aboveground part of *E. sibiricus* under combined salinity and drought stress, but MQ‐5 had the best and significant growth promoting effect; However, both MQ‐5 and GN‐1 had no significant effect on the fresh weight of the root of *E. sibiricus* under combined salinity and drought stress.

After treatment with MQ‐5, the average dry weight of aboveground part of *E. sibiricus* was increased by 18.62% (Figure 3C). Both MQ‐5 and GN‐1 could increase the aboveground dry weight of *E. sibiricus* under combined salinity and drought stress, but MQ‐5 had the best and significant growth‐promoting effect. However, both MQ‐5 and GN‐1 had no significant effect on root dry weight of *E. sibiricus* under combined salinity and drought stress.

#### 3.3.3 Determination of PGPR Growth Promoting Traits of *E. sibiricus*

When Salkowski's reagent was added to the fermentation broth of the IAA‐producing strain, the IAA produced turned to a pink substance. The value of OD530 after color development of the fermentation broth of each strain was recorded and the IAA yield of each strain could be calculated. Both GN‐1 and MQ‐5 can produce IAA; the IAA production of MQ‐5 was 8.24 mg/L and that of GN‐1 was 6.64 mg/L.

**FIGURE 4:**
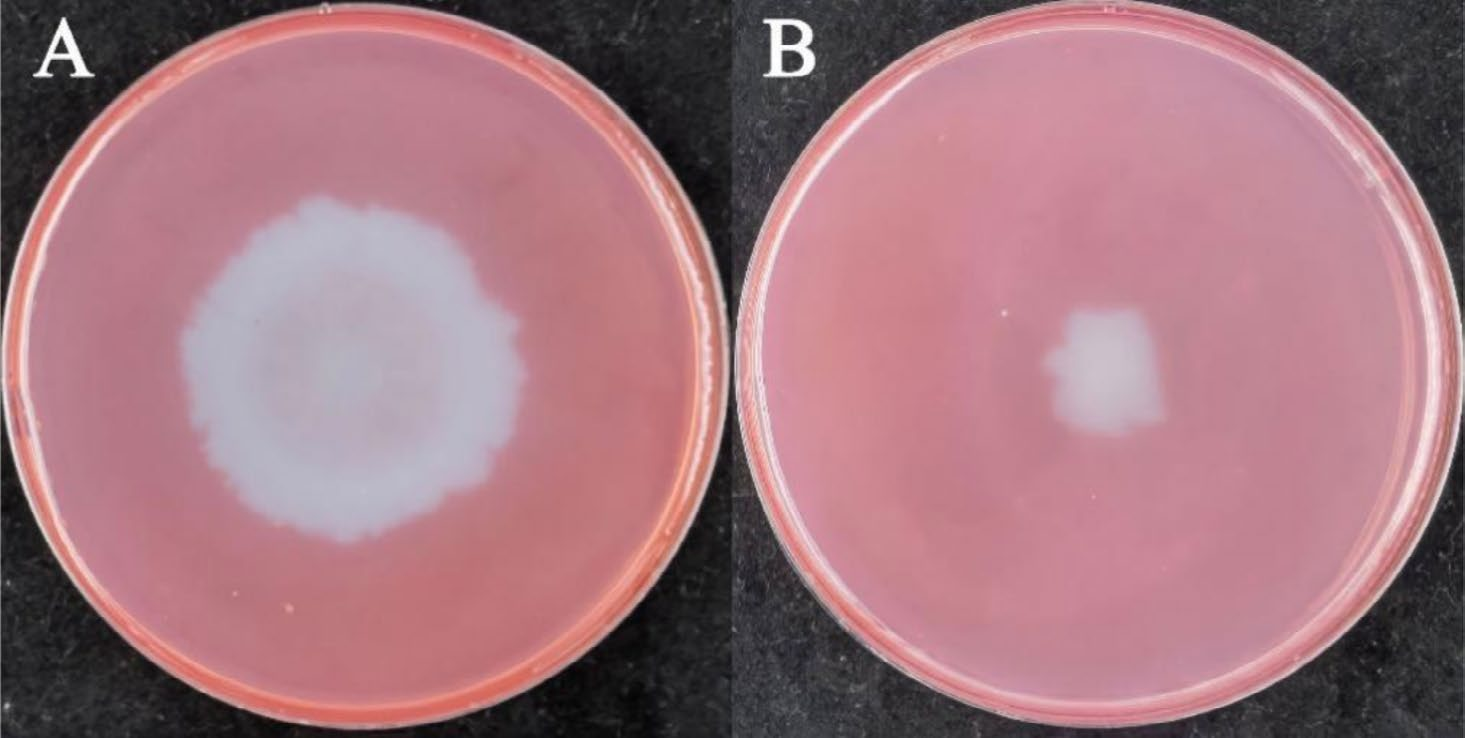
Degradation of cellulose by *E. sibiricus* PGPR. (A) the degradation result of GN‐1 on cellulose; (B) the degradation result of MQ‐5 on cellulose.

Both GN‐1 and MQ‐5 have the ability to degrade cellulose and form a cellulose degradation circle (Figure 4). The result is calculated by the following formula:

$$\mathrm{ADC}=\frac{D}{d}$$

The diameter of the colony was denoted as “*d*”, the diameter of the clear zone was denoted as “*D*”, and the ability to degrade cellulose was “ADC”. The ADC values of GN‐1 and MQ‐5 were 1.05 and 0.98, respectively.

**FIGURE 5:**
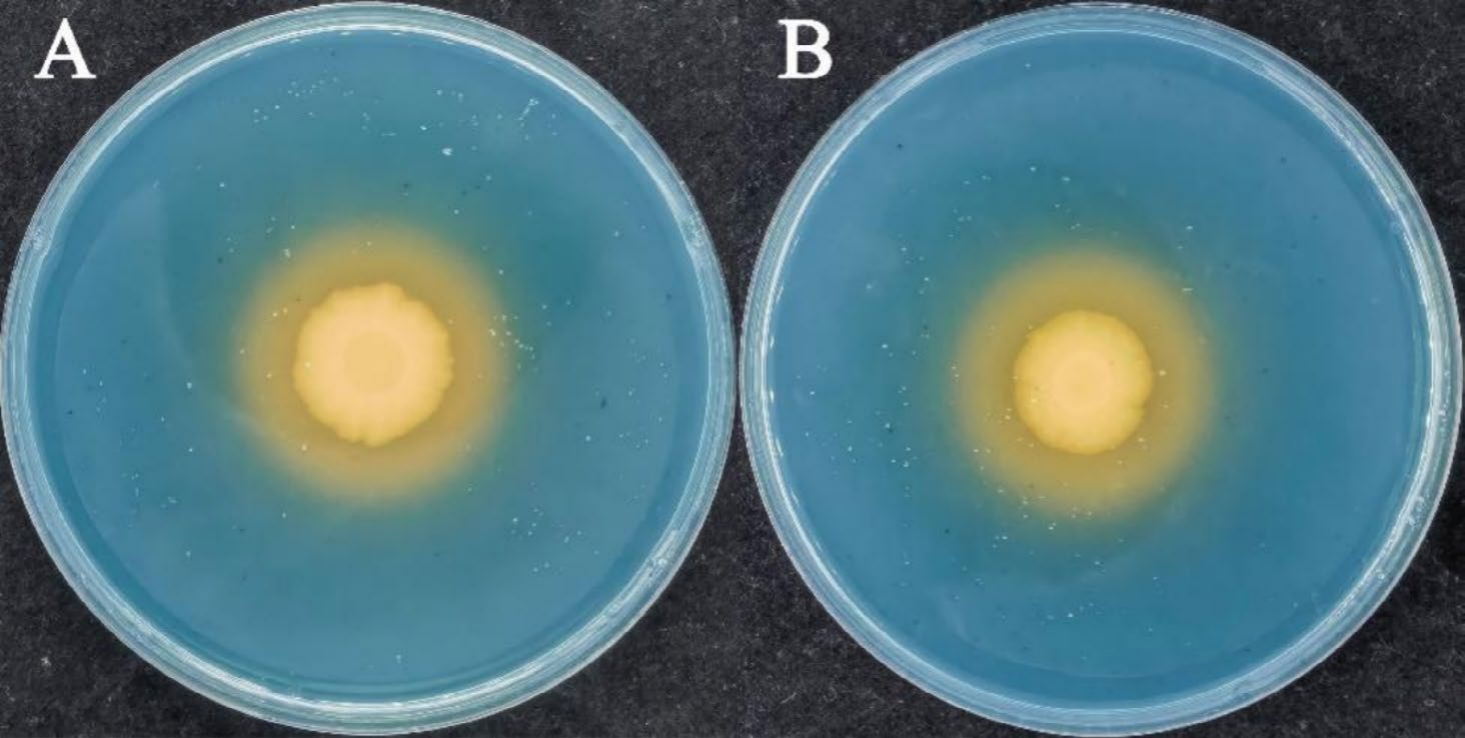
Siderophore production by *E. sibiricus* PGPR. (A) the siderophore producing effect of GN‐1; (B) the siderophore producing effect of MQ‐5.

Both GN‐1 and MQ‐5 have the ability to produce siderophores, and yellow halos appear around the colonies (Figure 5). The result is calculated by the following formula:

$$\mathrm{APS}=\frac{D}{d}$$

The diameter of the colony was denoted “*d*”, the diameter of the yellow circle was denoted “*D*”, and the ability to produce siderophores was “APS”. The APS values of GN‐1 and MQ‐5 for determining the ability to produce siderophores are 1.53 and 1.82, respectively. MQ‐5 has a greater ability to produce siderophores than GN‐1.

## 4 Discussion

Plant height, root length and aboveground and root fresh weight of *E. sibiricus* are important indicators to determine the growth promoting ability of PGPR, among which the plant height and aboveground biomass are particularly critical. Six strains including YJ‐5, MQ‐3, MQ‐5, GN‐1, GN‐2, and GN‐3 had good growth promoting effect on. *sibiricus* in the primary screening. The six strains were derived from saline alkaline soil collected in Zhangye, Gansu Province, soil collected in *E. sibiricus* grassland in Qinghai Plateau, and soil collected from Gannan grassland, Gansu Province. Similarly, Kerbab et al. (2021) reported that *B. atrophaeus* isolated from salinized soil can effectively improve the germination rate of wheat seeds and the growth of wheat seedlings under salinity stress; Li et al. (2022) reported that *B. zanthoxyli* and *B. altitudinis* isolated from the habitat of *Festuca elata* can effectively improve the seed germination rate, seedling tillering rate and leaf width of *F. elata* . It indicates that salinized soil samples and the native habitat of *E. sibiricus* are ideal sources of PGPR strains. The 6 PGPR strains isolated in this study belong to *Bacillus*, *Kocuria*, and *Brevibacillus*, respectively. Previous studies have shown that *Bacillus*, *Kocuria*, and *Brevibacillus* strains have plant growth promoting effects. Ali et al. (2022) reported that a *B*. *fungoides* strain with IAA, siderophore, ACC deaminase, exopolysaccharide and other growth promoting traits can alleviate the salinity stress of maize and significantly improve the plant height and root length; Goswami et al. (2014) reported that a strain of *K. turfanensis* 2 M4 with phosphorus solubilization, IAA production, siderophore and other growth promoting properties can significantly improve the plant height and biomass of peanut; Wang et al. (2022) reported that a *Brevibacillus* sp. with IAA‐producing ability can promote the lateral root growth of *Malus robusta* and improve the utilization rate of N, Zn, Fe, Cu, Mg, and other elements in leaves.

Six PGPR strains of *E. sibiricus* could alleviate the salinity stress of *E. sibiricus* , among which MQ‐5 was the most effective. This may be due to the fact that these strains increased their biomass under salt stress by secreting phytohormones such as IAA to promote growth and alleviate salt stress in *E. sibiricus* (Mansi and Gayatri 2021). Similarly, Sarwat et al. (2021) isolated several IAA‐producing *Bacillus* sp. strains from cotton rhizosphere soil, which can significantly improve the plant height and root length of cotton under salinity stress; Mahmood et al. (2016) reported that a *Pseudomonas fluorescens* with the ability to produce ACC deaminase, siderophore and IAA significantly increased the plant height of cucumber seedlings under salinity stress; Ma et al. (2024) reported that *Priestia* sp. Hl3, *Bacillus* HL6, and HG12, which have the ability to solubilize phosphate and produce ACC deaminase and IAA, promoted the biomass accumulation of alfalfa seedlings under salinity stress and improved the salinity tolerance of the plants.

In the pot experiment, both GN‐1 and MQ‐5 can both increase the average fresh weight and dry weight of aboveground parts and roots of *E. sibiricus* , which has a good growth promoting effect. Compared to GN‐1, MQ‐5 had a stronger growth promoting effect on the biomass of *E. sibiricus* ; However, GN‐1 and MQ‐5 had no significant effect on plant height and root length. This may be due to the fact that by the 35th day of harvest, the jointing stage of *E. sibiricus* plants has ended, the elongation of leaves and roots tends to slow down in the late growth stage, and the accumulation of biomass is mainly reflected in the increase of in fresh and dry weight of the plant.

The combined stress of salinity and drought significantly reduced the biomass of *E. sibiricus* , especially the fresh weight. Both GN‐1 and MQ‐5 treatments can alleviate the combined salinity and drought stress of *E. sibiricus* , which may be due to the involvement of GN‐1 and MQ‐5 in inducing the tolerance of *E. sibiricus* to abiotic stress (Abdelwahab et al. 2022). Khan et al. (2019) used *Bacillus* sp. isolated from chickpea rhizosphere soil to induce high accumulation of carbohydrates, riboflavin, L‐asparagine, etc. in the leaves of chickpea seedlings under drought stress and alleviate drought stress; Jochum et al. (2019) reported that *Enterobacter* sp. strain 16i with the ability to produce IAA and salicylic acid can significantly improve the root length, root surface area, and number of lateral roots of maize seedlings under drought stress; Barnawal et al. (2017) found that *B. subtilis* LDR2 treatment increased the IAA secretion of wheat seedlings and improved the drought resistance of wheat. Compared to GN‐1, MQ‐5 had a stronger growth‐promoting effect on the biomass of *E. sibiricus* under combined salinity and drought stress. This may be due to the fact that GN‐1 and MQ‐5 treatments alleviated the inhibition of combined salinity and drought stress on the elongation of *E. sibiricus* plants at the jointing stage and significantly increased the plant height compared to the control group; however, GN‐1 and MQ‐5 treatments had a weak alleviating effect on the stress on the root of *E. sibiricus* .

Both MQ‐5 and GN‐1 have good growth promoting effects on potted *E. sibiricus* , which may be due to the growth promoting functions of the 2 PGPR strains, such as IAA production, cellulose degradation and siderophore production. IAA is the most abundant and basic auxin in plants. It is a key regulator of plant stem and root development, lateral root formation and many other developmental processes (Coralie et al. 2022; Sun et al. 2018; Tsukanova et al. 2017). When IAA synthesized by the plant itself is insufficient, IAA produced by PGPR can further promote plant growth (Casanova‐Sáez and Voß 2019; Fernandes et al. 2022). Chebotar et al. (2022) applied a strain of *B. velezensis* capable of producing IAA to promote strawberry growth, which effectively improved leaf chlorophyll content, plant biomass and fruit yield.

Iron is an essential element to maintain plant life activities, and it is difficult for plants to directly use iron from soil. PGPR can promote iron dissolution through secreted siderophores, so it has become an ideal choice to alleviate iron deficiency in plants (Kobayashi et al. 2018; Lucena and Hernandez‐Apaolaza 2017; Tristan et al. 2021). The IAA production and siderophore capacity of MQ‐5 were better than those of GN‐1, which was consistent with the results that the growth‐promoting effect of MQ‐5 was better than that of GN‐1 in the pot experiment. Rehan et al. (2023) applied a variety of PGPR with the function of producing siderophores to promote the growth of tomato, which effectively improved the plant height, root length, and leaf area.

The cellulose‐degrading ability of PGPRs can accelerate the decomposition of plant debris in the soil, which helps nutrient cycling. Both MQ‐5 and GN‐1 showed cellulase activity. Benbrik et al. (2021) produced mixtures with cellulase activity using *Pseudomonas* sp., *Sphingobacterium suaedae*, *B. pumilus* , and *B. cereus* and used them to promote the growth of brassica, maize, and tomato, effectively increasing plant height and biomass and facilitating the uptake of phosphorus and potassium by the plants. In addition, PGPR contains multiple extracellular enzymes, such as cellulase, which may be involved in inhibiting soil‐borne pathogenic microorganisms. Karthika et al. (2020) reported that the *Bacillus* sp. strain KTMA4 can produce proteases, cellulases, lipases, xylanases, and *β*‐1,3‐glucanases. This strain was found to effectively inhibit the activities of Fusarium oxysporum and Alternaria solani and promote the growth of tomatoes.

## 5 Conclusion

In conclusion, two PGPR strains of *E. sibiricus* were obtained, GN‐1 and MQ‐5, both of which could both produce IAA and siderophore, and had the ability to degrade cellulose. They could increase the biomass of *E. sibiricus* in pot experiments and could alleviate salinity and drought stress. GN‐1 and MQ‐5 could be used to promote the growth of *E. sibiricus* under salinity and drought stress, and could solve the problems of slow growth rate and environmental stress of *E. sibiricus* . They are of great significance for the establishment of *E. sibiricus* grasslands in the Qinghai‐Tibet Plateau and have great development potential and application value.

## Funding

This work was supported by the Key Research and Development and Transformation Program of Qinghai Province of China (2022‐SF‐147) and the National Natural Science Foundation of China (32272538).

## Conflicts of Interest

The authors declare no conflicts of interest.

## Supporting information


**Appendix S1:** pei370106‐sup‐0001‐AppendixS1.docx.

## Data Availability

On reasonable request, the corresponding authors will provide the datasets created and/or analyzed during the current work.
