# Supplementary material for: Plant Growth‐Promoting Rhizobacteria Isolated From Natural Habitats Promote the Growth of Elymus sibiricus and Enhance Its Resistance to Abiotic Stress
Source: Plant Environ Interact. 2025 Dec 26;6(6):e70106. doi: 10.1002/pei3.70106 (PMC12741628; doi:10.1002/pei3.70106)
Supplement: Supplementary file 1 — Appendix S1: pei370106‐sup‐0001‐AppendixS1.docx. [file PEI3-6-e70106-s001.docx]

**Supplementary** **Materials**

**Plant Growth-Promoting Rhizobacteria Isolated from Natural Habitats Promote the Growth of *Elymus sibiricus* and Enhances Its Resistance to Abiotic Stress**

Ruiqi Liang^a^, Yunfei Xu^a^, Mengying Xu^a^, Lei Pan^a^, Zengmiao Lin^a^, Xikai Wang^a^, Weixia Wang^b^, Tingheng Zhu^a^*

^a^ College of Biotechnology and Bioengineering, Zhejiang University of Technology, Hangzhou 310014, Zhejiang Province, China

^b^ China National Rice Research Institute, Hangzhou 310006, Zhejiang Province, China

*Corresponding author: Tingheng Zhu

College of Biotechnology and Bioengineering, Zhejiang University of Technology, Hangzhou 310014, Zhejiang Province, China

[thzhu@zjut.edu.cn](mailto:thzhu@zjut.edu.cn)

Supporting Information Includes:

- 2 tables

- 7 figures


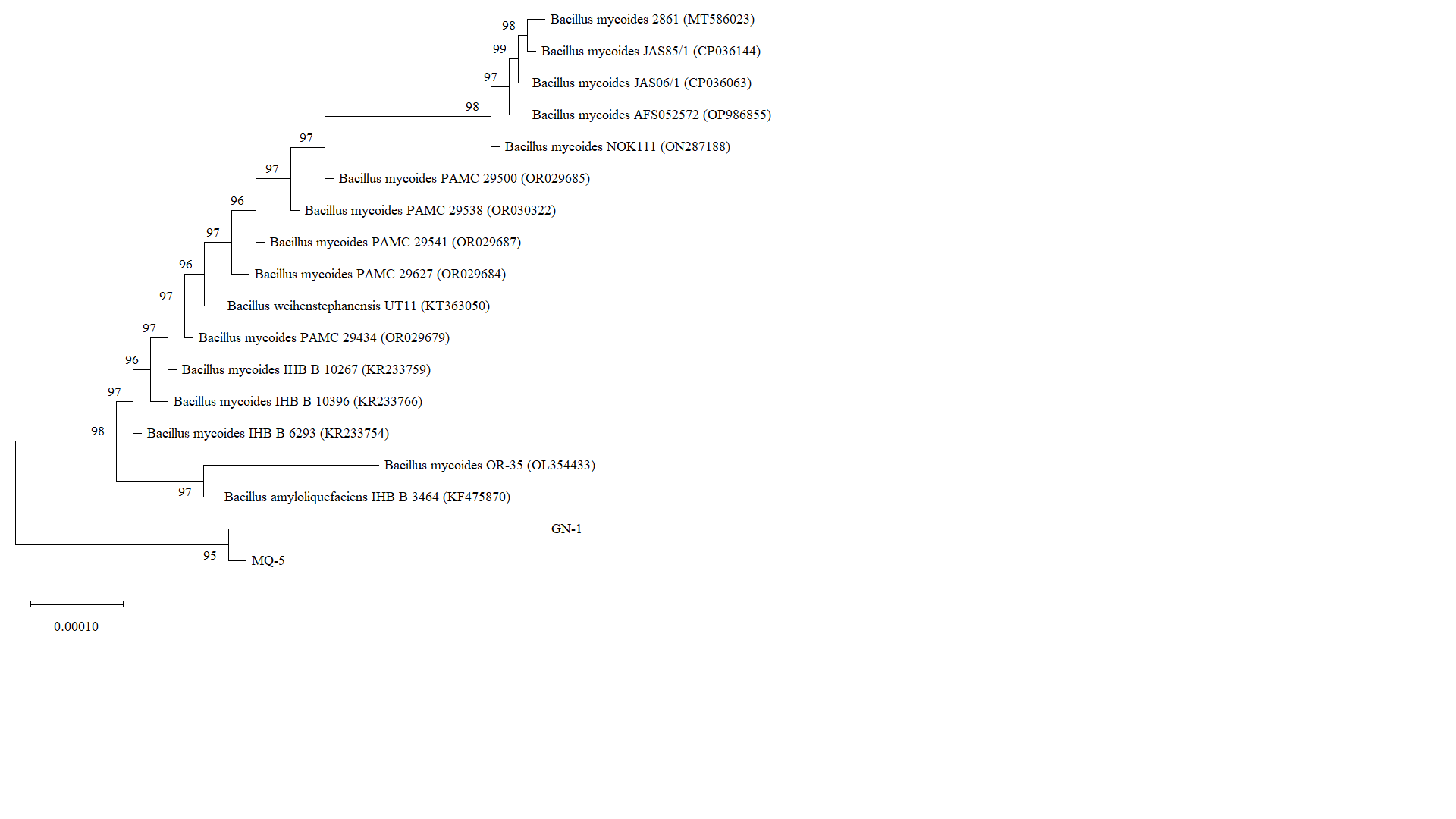


Fig S1 Phylogenetic tree based on 16S rRNA of PGPR of *Elymus sibiricus* GN-1 and MQ-5

Fig S2 Effect of PGPR on plant height and root length of *E. sibiricus* under salinity stress. *: p＜0.05; **: p＜0.01; ***: p＜0.001; No mark: not significant.

Fig S3 Effect of PGPR on fresh weight of aboveground parts and roots of *E. sibiricus* under salinity stress. *: p＜0.05; **: p＜0.01; no mark: not significant.

Fig S4 Effect of PGPR on plant height and root length of *E. sibiricus* under drought stress. *: p＜0.05; no mark: not significant.

Fig S5 Effect of PGPR on fresh weight of aboveground parts and roots of *E. sibiricus* under drought stress. No mark: not significant.

Fig S6 Effect of PGPR on plant height and root length of *E. sibiricus* under salinity and drought stress. No mark: not significant.

Fig S7 Effect of PGPR on fresh weight of aboveground parts and roots of *E. sibiricus* under salinity and drought stress. No mark: not significant.


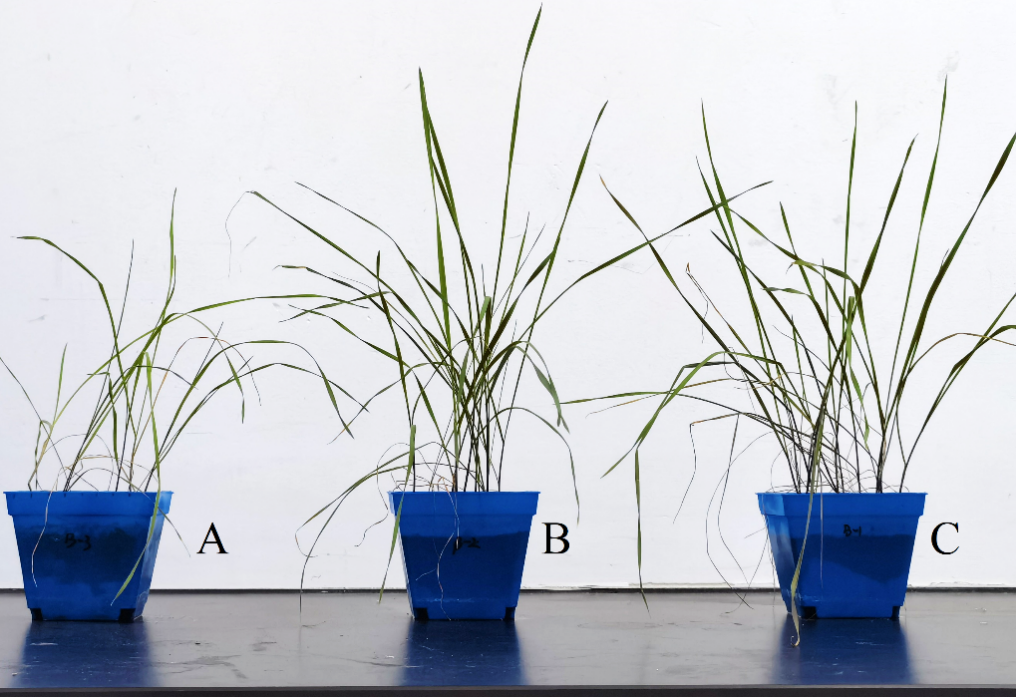


Fig. S8 Effect of PGPR on *E. sibiricus* in pot experiment. (A) control group, (B) GN-1 treatment group, (C) MQ-5 treatment group.

Table S1 16S rRNA gene PCR reaction system

| Ingredient | Volume (μL) |
| --- | --- |
| DNA template | 1 |
| 27F | 1 |
| 1492R | 1 |
| Phanta Master Mix | 25 |
| ddH_2_O | 22 |
| Total Volume | 50 |

Table S2 16S rRNA gene PCR reaction program

| Step | Temperature（℃） | Time |
| --- | --- | --- |
| 1 | 95 | 5 min |
| 2 | 95 | 15 s |
| 3 | 57 | 15 s |
| 4 | 72 | 4 min |
| 5 | Return to step 2 | 30 cycles |
| 6 | 72 | 5min |
| 7 | 16 | - |

Table S3 16S rRNA gene sequences of PGPR

| Strain | 16S rRNA gene sequence |
| --- | --- |
| YJ-5 | GGCGGCGTGCTTAACACATGCAAGTCGAACGATGATGCCCAGCTTGCTGGGCGGATTAGTGGCGAACGGGTGAGTAATACGTGAGTAACCTGCCCTTGACTCTGGGATAAGCCTGGGAAACTGGGTCTAATACTGGATACTACGTCCCATCGCATGGTGGGGGGTGGAAAGGGTTTTACTGGTTTTGGATGGGCTCACGGCCTATCAGCTTGTTGGTGGGGTAATGGCTCACCAAGGCGACGACGGGTAGCCGGCCTGAGAGGGTGACCGGCCACACTGGGACTGAGACACGGCCCAGACTCCTACGGGAGGCAGCAGTGGGGAATATTGCACAATGGGCGGAAGCCTGATGCAGCGACGCCGCGTGAGGGATGACGGCCTTCGGGTTGTAAACCTCTTTCAGTAGGGAAGAAGCGAGAGTGACGGTACCTGCAGAAGAAGCGCCGGCTAACTACGTGCCAGCAGCCGCGGTAATACGTAGGGCGCAAGCGTTGTCCGGAATTATTGGGCGTAAAGAGCTCGTAGGCGGTTTGTCGCGTCTGCTGTGAAAGCCCGGGGCTCAACCCCGGGTCTGCAGTGGGTACGGGCAGACTAGAGTGCAGTAGGGGAGACTGGAATTCCTGGTGTAGCGGTGAAATGCGCAGATATCAGGAGGAACACCGATGGCGAAGGCAGGTCTCTGGGCTGTTACTGACGCTGAGGAGCGAAAGCATGGGGAGCGAACAGGATTAGATACCCTGGTAGTCCATGCCGTAAACGTTGGGCACTAGGTGTGGGGGACATTCCACGTTCTCCGCGCCGTAGCTAACGCATTAAGTGCCCCGCCTGGGGAGTACGGCCGCAAGGCTAAAACTCAAAGGAATTGACGGGGGCCCGCACAAGCGGCGGAGCATGCGGATTAATTCGATGCAACGCGAAGAACCTTACCAAGGCTTGACATTCACCGGATCGCCCCAGAGATGGGGTTTCCCTTCGGGGTCGGTGGACAGGTGGTGCATGGTTGTCGTCAGCTCGTGTCGTGAGATGTTGGGTTAAGTCCCGCAACGAGCGCAACCCTCGTTCTATGTTGCCAGCACGTGATGGTGGGGACTCATAGGAGACTGCCGGGGTCAACTCGGAGGAAGGTGGGGATGACGTCAAATCATCATGCCCCTTATGTCTTGGGCTTCACGCATGCTACAATGGCCGGTACAAAGGGTTGCGATACTGTGAGGTGGAGCTAATCCCAAAAAGCCGGTCTCAGTTCGGATTGAGGTCTGCAACTCGACCTCATGAAGTCGGAGTCGCTAGTAATCGCAGATCAGCAACGCTGCGGTGAATACGTTCCCGGGCCTTGTACACACCGCCCGTCAAGTCACGAAAGTTGGTAACACCCGAAGCCGGTGGCCTAACCCCTTTGTGGGAGGGAGCCGTCGAAGGTGGGACTGGCGATTGGGACTAAGTCGTACAGGG |
| MQ-3 | CCAATCATCTGTCCCACCTTAGGCGGCTGGCTCCATAAAGGTTACCCCACCGACTTCGGGTGTTACAAACTCTCGTGGTGTGACGGGCGGTGTGTACAAGGCCCGGGAACGTATTCACCGCGGCATGCTGATCCGCGATTACTAGCGATTCCAGCTTCATGTAGGCGAGTTGCAGCCTACAATCCGAACTGAGAACGGTTTTATGAGATTAGCTCCACCTCGCGGTCTTGCAGCTCTTTGTACCGTCCATTGTAGCACGTGTGTAGCCCAGGTCATAAGGGGCATGATGATTTGACGTCATCCCCACCTTCCTCCGGTTTGTCACCGGCAGTCACCTTAGAGTGCCCAACTTAATGATGGCAACTAAGATCAAGGGTTGCGCTCGTTGCGGGACTTAACCCAACATCTCACGACACGAGCTGACGACAACCATGCACCACCTGTCACTCTGCTCCCGAAGGAGAAGCCCTATCTCTAGGGTTTTCAGAGGATGTCAAGACCTGGTAAGGTTCTTCGCGTTGCTTCGAATTAAACCACATGCTCCACCGCTTGTGCGGGCCCCCGTCAATTCCTTTGAGTTTCAGCCTTGCGGCCGTACTCCCCAGGCGGAGTGCTTAATGCGTTAACTTCAGCACTAAAGGGCGGAAACCCTCTAACACTTAGCACTCATCGTTTACGGCGTGGACTACCAGGGTATCTAATCCTGTTTGCTCCCCACGCTTTCGCGCCTCAGTGTCAGTTACAGACCAGAAAGTCGCCTTCGCCACTGGTGTTCCTCCATATCTCTACGCATTTCACCGCTACACATGGAATTCCACTTTCCTCTTCTGCACTCAAGTCTCCCAGTTTCCAATGACCCTCCACGGTTGAGCCGTGGGCTTTCACATCAGACTTAAGAAACCACCTGCGCGCGCTTTACGCCCAATAATTCCGGATAACGCTTGCCACCTACGTATTACCGCGGCTGCTGGCACGTAGTTAGCCGTGGCTTTCTGGTTAGGTACCGTCAAGGTGCCAGCTTATTCAACTAGCACTTGTTCTTCCCTAACAACAGAGTTTTACGACCCGAAAGCCTTCATCACTCACGCGGCGTTGCTCCGTCAGACTTTCGTCCATTGCGGAAGATTCCCTACTGCTGCCTCCCGTAGGAGTCTGGGCCGTGTCTCAGTCCCAGTGTGGCCGATCACCCTCTCAGGTCGGCTACGCATCGTTGCCTTGGTGAGCCGTTACCTCACCAACTAGCTAATGCGACGCGGGTCCATCCATAAGTGACAGCCGAAGCCGCCTTTCAATTTCGAACCATGCAGTTCAAAATGTTATCCGGTATTAGCCCCGGTTTCCCGGAGTTATCCCAGTCTTATGGGCAGGTTACCCACGTGTTACTCACCCGTCCGCCGCTAACTTCTTGAGAGCAAGCTCTCAATCCATTCGCTCGACTTGCATGTATTAGGACGCGCA |
| MQ-5 | GCGGCGTCCTAATACATGCAAGTCGAGCGAATGGATTAAGAGCTTGCTCTTATGAAGTTAGCGGCGGACGGGTGAGTAACACGTGGGTAACCTACCCATAAGACTGGGATAACTCCGGGAAACCGGGGCTAATACCGGATAATATTTTGAACTGCATAGTTCGAAATTGAAAGGCGGCTTCGGCTGTCACTTATGGATGGACCCGCGTCGCATTAGCTAGTTGGTGAGGTAACGGCTCACCAAGGCGACGATGCGTAGCCGACCTGAGAGGGTGATCGGCCACACTGGGACTGAGACACGGCCCAGACTCCTACGGGAGGCAGCAGTAGGGAATCTTCCGCAATGGACGAAAGTCTGACGGAGCAACGCCGCGTGAGTGATGAAGGCTTTCGGGTCGTAAAACTCTGTTGTTAGGGAAGAACAAGTGCTAGTTGAATAAGCTGGCACCTTGACGGTACCTAACCAGAAAGCCACGGCTAACTACGTGCCAGCAGCCGCGGTAATACGTAGGTGGCAAGCGTTATCCGGAATTATTGGGCGTAAAGCGCGCGCAGGTGGTTTCTTAAGTCTGATGTGAAAGCCCACGGCTCAACCGTGGAGGGTCATTGGAAACTGGGAGACTTGAGTGCAGAAGAGGAAAGTGGAATTCCATGTGTAGCGGTGAAATGCGTAGAGATATGGAGGAACACCAGTGGCGAAGGCGACTTTCTGGTCTGTAACTGACACTGAGGCGCGAAAGCGTGGGGAGCAAACAGGATTAGATACCCTGGTAGTCCACGCCGTAAACGATGAGTGCTAAGTGTTAGAGGGTTTCCGCCCTTTAGTGCTGAAGTTAACGCATTAAGCACTCCGCCTGGGGAGTACGGCCGCAAGGCTGAAACTCAAAGGAATTGACGGGGGCCCGCACAAGCGGTGGAGCATGTGGTTTAATTCGAAGCAACGCGAAGAACCTTACCAGGTCTTGACATCCTCTGAAAACTCTAGAGATAGAGCTTCTCCTTCGGGAGCAGAGTGACAGGTGGTGCATGGTTGTCGTCAGCTCGTGTCGTGAGATGTTGGGTTAAGTCCCGCAACGAGCGCAACCCTTGATCTTAGTTGCCATCATTAAGTTGGGCACTCTAAGGTGACTGCCGGTGACAAACCGGAGGAAGGTGGGGATGACGTCAAATCATCATGCCCCTTATGACCTGGGCTACACACGTGCTACAATGGACGGTACAAAGAGCTGCAAGACCGCGAGGTGGAGCTAATCTCATAAAACCGTTCTCAGTTCGGATTGTAGGCTGCAACTCGCCTACATGAAGCTGGAATCGCTAGTAATCGCGGATCAGCATGCCGCGGTGAATACGTTCCCGGGCCTTGTACACACCGCCCGTCACACCACGAGAGTTTGTAACACCCGAAGTCGGTGGGGTAACCTTTATGGAGCCAGCCGCCTAAGGTGGGACAGATGATTGGGGTGAAGTCGTACAGG |
| GN-1 | TGCGGCGTGCCTAATACATGCAAGTCGAGCGAATGGATTAAGAGCTTGCTCTTATGAAGTTAGCGGCGGACGGGTGAGTAACACGTGGGTAACCTACCCATAAGACTGGGATAACTCCGGGAAACCGGGGCTAATACCGGATAATATTTTGAACTGCATAGTTCGAAATTGAAAGGCGGCTTCGGCTGTCACTTATGGATGGACCCGCGTCGCATTAGCTAGTTGGTGAGGTAACGGCTCACCAAGGCGACGATGCGTAGCCGACCTGAGAGGGTGATCGGCCACACTGGGACTGAGACACGGCCCAGACTCCTACGGGAGGCAGCAGTAGGGAATCTTCCGCAATGGACGAAAGTCTGACGGAGCAACGCCGCGTGAGTGATGAAGGCTTTCGGGTCGTAAAACTCTGTTGTTAGGGAAGAACAAGTGCTAGTTGAATAAGCTGGCACCTTGACGGTACCTAACCAGAAAGCCACGGCTAACTACGTGCCAGCAGCCGCGGTAATACGTAGGTGGCAAGCGTTATCCGGAATTATTGGGCGTAAAGCGCGCGCAGGTGGTTTCTTAAGTCTGATGTGAAAGCCCACGGCTCAACCGTGGAGGGTCATTGGAAACTGGGAGACTTGAGTGCAGAAGAGGAAAGTGGAATTCCATGTGTAGCGGTGAAATGCGTAGAGATATGGAGGAACACCAGTGGCGAAGGCGACTTTCTGGTCTGTAACTGACACTGAGGCGCGAAAGCGTGGGGAGCAAACAGGATTAGATACCCTGGTAGTCCACGCCGTAAACGATGAGTGCTAAGTGTTAGAGGGTTTCCGCCCTTTAGTGCTGAAGTTAACGCATTAAGCACTCCGCCTGGGGAGTACGGCCGCAAGGCTGAAACTCAAAGGAATTGACGGGGGCCCGCACAAGCGGTGGAGCATGTGGTTTAATTCGAAGCAACGCGAAGAACCTTACCAGGTCTTGACATCCTCTGAAAACTCTAGAGATAGAGCTTCTCCTTCGGGAGCAGAGTGACAGGTGGTGCATGGTTGTCGTCAGCTCGTGTCGTGAGATGTTGGGTTAAGTCCCGCAACGAGCGCAACCCTTGATCTTAGTTGCCATCATTAAGTTGGGCACTCTAAGGTGACTGCCGGTGACAAACCGGAGGAAGGTGGGGATGACGTCAAATCATCATGCCCCTTATGACCTGGGCTACACACGTGCTACAATGGACGGTACAAAGAGCTGCAAGACCGCGAGGTGGAGCTAATCTCATAAAACCGTTCTCAGTTCGGATTGTAGGCTGCAACTCGCCTACATGAAGCTGGAATCGCTAGTAATCGCGGATCAGCATGCCGCGGTGAATACGTTCCCGGGCCTTGTACACACCGCCCGTCACACCACGAGAGTTTGTAACACCCGAAGTCGGTGGGGTAACCTTTATGGAGCCAGCCGCCTAAGGTGGGACAGATGATTGGGGTGA |
| GN-2 | CGGCGTGCCTAATACATGCAAGTCGAGCGGACAGATGGGAGCTTGCTCCCTGATGTTAGCGGCGGACGGGTGAGTAACACGTGGGTAACCTGCCTGTAAGACTGGGATAACTCCGGGAAACCGGGGCTAATACCGGATGCTTGTTTGAACCGCATGGTTCAAACATAAAAGGTGGCTTCGGCTACCACTTACAGATGGACCCGCGGCGCATTAGCTAGTTGGTGAGGTAATGGCTCACCAAGGCAACGATGCGTAGCCGACCTGAGAGGGTGATCGGCCACACTGGGACTGAGACACGGCCCAGACTCCTACGGGAGGCAGCAGTAGGGAATCTTCCGCAATGGACGAAAGTCTGACGGAGCAACGCCGCGTGAGTGATGAAGGTTTTCGGATCGTAAAGCTCTGTTGTTAGGGAAGAACAAGTACCGTTCGAATAGGGCGGTACCTTGACGGTACCTAACCAGAAAGCCACGGCTAACTACGTGCCAGCAGCCGCGGTAATACGTAGGTGGCAAGCGTTGTCCGGAATTATTGGGCGTAAAGGGCTCGCAGGCGGTTTCTTAAGTCTGATGTGAAAGCCCCCGGCTCAACCGGGGAGGGTCATTGGAAACTGGGGAACTTGAGTGCAGAAGAGGAGAGTGGAATTCCACGTGTAGCGGTGAAATGCGTAGAGATGTGGAGGAACACCAGTGGCGAAGGCGACTCTCTGGTCTGTAACTGACGCTGAGGAGCGAAAGCGTGGGGAGCGAACAGGATTAGATACCCTGGTAGTCCACGCCGTAAACGATGAGTGCTAAGTGTTAGGGGGTTTCCGCCCCTTAGTGCTGCAGCTAACGCATTAAGCACTCCGCCTGGGGAGTACGGTCGCAAGACTGAAACTCAAAGGAATTGACGGGGGCCCGCACAAGCGGTGGAGCATGTGGTTTAATTCGAAGCAACGCGAAGAACCTTACCAGGTCTTGACATCCTCTGACAATCCTAGAGATAGGACGTCCCCTTCGGGGGCAGAGTGACAGGTGGTGCATGGTTGTCGTCAGCTCGTGTCGTGAGATGTTGGGTTAAGTCCCGCAACGAGCGCAACCCTTGATCTTAGTTGCCAGCATTCAGTTGGGCACTCTAAGGTGACTGCCGGTGACAAACCGGAGGAAGGTGGGGATGACGTCAAATCATCATGCCCCTTATGACCTGGGCTACACACGTGCTACAATGGACAGAACAAAGGGCAGCGAAACCGCGAGGTTAAGCCAATCCCACAAATCTGTTCTCAGTTCGGATCGCAGTCTGCAACTCGACTGCGTGAAGCTGGAATCGCTAGTAATCGCGGATCAGCATGCCGCGGTGAATACGTTCCCGGGCCTTGTACACACCGCCCGTCACACCACGAGAGTTTGTAACACCCGAAGTCGGTGAGGTAACCTTTTAGGAGCCAGCCGCCGAAGGTGGGACAGATGATTGGGGTGAAGT |
| GN-3 | CTAATACATGCAAGTCGAGCGAGGGTCTTCGGACCCTAGCGGCGGACGGGTGAGTAACACGTAGGCAACCTGCCTGTAAGACTGGGATAACATAGGGAAACTTATGCTAATACCGGATAGGGTTTTGCTTCTCCTGAAGCGAAACGGAAAGATGGCGTAAGCTATCACTTGCAGATGGGCCTGCGGCGCATTAGCTAGTTGGTGAGGTAATGGCTCACCAAGGCGACGATGCGTAGCCGACCTGAGAGGGTGACCGGCCACACTGGGACTGAGACACGGCCCAGACTCCTACGGGAGGCAGCAGTAGGGAATTTTCCACAATGGACGAAAGTCTGATGGAGCAACGCCGCGTGAACGATGAAGGCTTTCGGGTCGTAAAGTTCTGTTGTTAGGGAAGAAACAGTGCCATTTAAATAAGGTGGCACCTTGACGGTACCTAACGAGAAAGCCACGGCTAACTACGTGCCAGCAGCCGCGGTAATACGTAGGTGGCAAGCGTTGTCCGGAATTATTGGGCGTAAAGCGCGCGCAGGTGGCTATGTAAGTCTGATGTTAAAGCCCGAGGCTCAACCTCGGTTCGCATTGGAAACTGTGTAGCTTGAGTGCAGGAGAGGAAAGTGGTATTCCACGTGTAGCGGTGAAATGCGTAGAGATGTGGAGGAACACCAGTGGCGAAGGCGACTTTCTGGCCTGTAACTGACACTGAGGCGCGAAAGCGTGGGGAGCAAACAGGATTAGATACCCTGGTAGTCCACGCCGTAAACGATGAGTGCTAGGTGTTAGGGGTTTCAATACCCTTAGTGCCGCAGCTAACGCAATAAGCACTCCGCCTGGGGAGTACGCTCGCAAGAGTGAAACTCAAAGGAATTGACGGGGGCCCGCACAAGCGGTGGAGCATGTGGTTTAATTCGAAGCAACGCGAAGAACCTTACCAGGTCTTGACATCCCACTGACCGCTCTAGAGATAGAGCTTCCCTTCGGGGCAGTGGTGACAGGTGGTGCATGGTTGTCGTCAGCTCGTGTCGTGAGATGTTGGGTTAAGTCCCGCAACGAGCGCAACCCTTATCTTTAGTTGCCAGCATTCAGTTGGGCACTCTAGAGAGACTGCCGTCGACAAGACGGAGGAAGGCGGGGATGACGTCAAATCATCATGCCCCTTATGACCTGGGCTACACACGTGCTACAATGGTTGGTACAACGGGATGCTACTTCGCGAGAAGATGCTAATCTCTTAAAACCAATCTCAGTTCGGATTGTAGGCTGCAACTCGCCTACATGAAGTCGGAATCGCTAGTAATCGCGGATCAGCATGCCGCGGTGAATACGTTCCCGGGCCTTGTACACACCGCCCGTCACACCACGGGAGTTTGCAACACCCGAAGTCGGTGAGGTAACCGCAAGGAGCCAGCCGCCGAAGGTGGGGTAGATAACTGGGGTG |
